# Clinical outcome analysis of intramural myoma greater than 8 cm in diameter removed during caesarean section: a retrospective study

**DOI:** 10.1186/s12905-023-02210-9

**Published:** 2023-02-11

**Authors:** Chunbo Shi, Jinliang Chen, Aner Chen

**Affiliations:** 1 Department of Obstetrics and Gynecology, Ningbo Women and Children’s Hospital, Ningbo, 315012 Zhejiang China; 2 Radiology Department, Ningbo Women and Children’s Hospital, Ningbo, 315012 Zhejiang China

**Keywords:** Caesarean myomectomy, Subserosal myomectomy, Endometrial myomectomy

## Abstract

**Objective:**

To explore the safety and efficiency of endometrial myomectomy (EM) and Serosal myomectomy (SM) for the removal of intramural myoma greater than 8 cm in diameter during cesarean section.

**Methods:**

Retrospective analysis and follow-up were used, and 190 cases of pregnancy complicated with uterine myoma from Jan. 2017 to May 2022 in Ningbo Women’s and Children’s Hospital were collected, 130 cases of caesarean myomectomy as study group, 64 cases of EM as study group A, 66 cases of SM as study group B, 33 cases with uterine fibroids removed before suturing the uterine incision as study group B1, 33 cases with uterine incision sutured followed by removal of fibroids as study group B2, 60 cases of Caesarean section alone as control group. To compare perioperative conditions between and within groups.

**Results:**

① Operation time, postoperative exhaust time, pre- and post-operative haemoglobin drop, intraoperative blood loss were all more than those of the control group in the study group (68.65 ± 11.87 vs 56.17 ± 9.18 min, 21.04 ± 4.98 vs 17.03 ± 1.3 h, 1.27 ± 0.59 vs 1.09 ± 0.43 g/dl, 613 ± 221 vs 532 ± 156 ml, *P* < 0.001, *P* < 0.001, *P* = 0.025, *P* = 0.011). ② For type III and V fibroids, the time of myoma removal, postoperative exhaust and pre- and post-operative haemoglobin drop and intraoperative blood loss in study group A were less than those in study group B (18.02 ± 3.89 vs 20.19 ± 5.32 min, 18.83 ± 2.57 vs 23.93 ± 6.84 h, 600 ± 194 vs 730 ± 277 ml, 1.20 ± 0.57 vs 1.59 ± 0.70 g/dl, *P* = 0.036, *P* < 0.001, *P* = 0.014, *P* = 0.008); For type IV uterine fibroids, only postoperative exhaust time was less in Study Group A than in Study Group B (19.27 ± 2.2 vs 21.35 ± 3.23 h, *P* = 0.016). ③ Time of myoma removed was less in study group B1 than in study group B2 (18.24 ± 4.53 vs 20.7 ± 4.59 min, *P* = 0.033).

**Conclusion:**

It is safe and feasible to remove interstitial myomas larger than 8 cm in diameter during caesarean section. EM has the advantage of shorter operation time and less intraoperative bleeding, SM, in a way that the myoma is removed before suturing the uterine incision, can shorten the myomectomy time. It can benefit the patients more.

## Introduction

Uterine myomas are the most common benign tumours in the female reproductive organs and affect 20% to 40% of women at reproductive age [1]. The risk of uterine myoma during pregnancy is 0.3–0.5% [2]. As the three-child policy has been implemented in China, the age of pregnancy has increased and assisted reproductive technologies have been developed, the rate of pregnancy combined with uterine fibroids has also increased [3]. The risk of abortion, preterm delivery, fetal growth restriction, placenta praevia and postpartum haemorrhage is increased due to the pregnancy combined with uterine myoma [4]. There is still a controversy over whether to remove fibroids at the same time as a caesarean section [5, 6]. Traditional approach is usually against caesarean myomectomy. The major disadvantage is the risk of intractable hemorrhage [7] which could be attributed to the increased perfusion of uterus during pregnancy. The subsequent risk of reoperation, transfusion, ligation or arterial embolization, and even hysterectomy may be increased [8, 9]. However, in recent years, a lot of clinical studies have confirmed the safety and feasibility of myomectomy during cesarean delivery, especially for fibroids less than 8 cm in diameter [10, 11]. But the safety of removing intramural myoma larger than 8 cm in diameter during the caesarean section has rarely been reported. The following is a summary of 130 cases of caesarean myomectomy by improved surgical techniques for type 3 to 5 uterine fibroids (FIGO) [12] greater than 8 cm in diameter, performed by the author’s medical team.

## Materials and methods

The investigation was carried on 190 cases of pregnant women diagnosed for uterine fibroids greater than 8 cm in diameter during pregnancy and scheduled for cesarean section (CS) between Jan. 2017 to May 2022 at Ningbo Women and Children’s Hospital were retrospectively reviewed in this study. The Institutional Review Board of Ningbo Women and Children’s approved this study, and the need for informed consent was waived because of the retrospective nature of this study.

Electronic medical records were reviewed to collect patient demographics, obstetrical history, operative reports, and clinical notes. The inclusion criteria were preoperative ultrasound and intraoperative diagnosed uterine fibroids of type (3–5) according to FIGO classification and fibroids larger than 8 cm in maximum diameter. The following patients were excluded: Patients in high risk of postpartum haemorrhage such as twins, placenta praevia, placenta implantation, placenta abruption, coagulation disorders etc. 130 women who had consented to and undergone caesarean myomectomy were assigned to the study group, while the other 60 women were assigned to the control group. The Study Group included 64 women undergone EM who were assigned to the Study GroupA, 66 women undergone SM who were assigned to the Study GroupB. Study Group B included Study Group B1 thirty-three cases who removed uterine fibroids before suturing the uterine incision and Study Group B2 thirty-three cases who sutured the uterine incision followed by myomectomy.

All patients were prepared for autologous blood transfusion before the operation. Cesarean section was performed with a midline abdominal incision and spinal anesthesia. Following the delivery of the newborn and placenta, intravenous carbetocin 0.1 mg was injected. Then the uterus was delivered out of the abdominal cavity and the lower uterine was tied with a tourniquet. Study Group A and Study Group B1 removed uterine fibroids before suturing the uterine incision and Study group B2 sutured the uterine incision followed by myomectomy. Myomectomy process: The assistant squeezes the myoma towards the endometrium or serosa, then a linear incision at the most prominent part of the myoma was made by electro scalpel till fibroid surface via the myoma pseudocapsule. Surgeon then extracts the fibroid by progressive tractions, while sutuer-ligating the vessels at the basal part to provide hemosta, finally closing tumor cavity using continuous suture method with absorbable 1 Polysorb CL-905, suturing the serosal or endometrium with 2–0 Vicryl. Observe the amount of uterine bleeding after loosening the tourniquet. If the bleeding was heavy, medications were administered as the initial form of conservative treatment. Intrauterine injections of Carboprost-Tromethamine 250 mg and intravenous tranexamic acid 1 g are used to enhance uterine contraction. The effect is not satisfactory, thus more hemostatic measures are added. If the uterine incision is not sutured, the preferred method is to tamp down the uterine cavity with a balloon and, if necessary, ligate the ascending branches of the uterine artery; If the uterine incision has been sutured, it is best to ligate one or both ascending branches of the uterine artery. If necessary, a balloon is inflated into the uterine cavity through the vagina.

The surgical outcomes that included duration of surgery, blood loss (volume method + weighing gauze), preoperative and postoperative hemoglobin values and surgical complications (such as postpartum haemorrhage, transfusion, uterine artery ligation, uterine stuffing, postoperative fever (temperature ≥ 38 °C), intrauterine adhesion) were recorded for statistical analysis.

Statistics Statistical analysis was performed using SPSS 23.0 software. Data were presented as mean ± SD, median (min, max) or count (percentage). Comparison between groups was determined by t test, one-way ANOVA test, Wilcoxon rank sum test or chi-square test. *P* < 0.05 was considered significant.

## Result

The study groupA, the study group Band the control groups reached the number of 190 enrolled and analyzed patients. All groups were statistically similar for age, gravidity, parity, gestational age at delivery, number of fibroids, myoma size, myoma location and indications (*P* > 0.05 for all) (Table 1).

**Table 1:** Baseline characteristics of patients

	Study group A (n = 64)	Study group B (n = 66)	Control group (n = 60)	*P*-value
Age (year)	33.83 ± 5.10	33.36 ± 4.80	33.58 ± 5.04	> 0.05
Gravidity	2 (1,4)	2 (1,7)	2 (1,7)	> 0.05
Parity	0 (0,2)	0 (0,1)	0 (0,1)	> 0.05
Gestational age (week)	37.73 ± 1.33	37.91 ± 1.17	37.7 ± 1.45	> 0.05
Size of largest myoma (cm)	9 (8,15)	9 (8,17)	9 (8,18)	> 0.05
Number of myomas	1 (1,5)	2 (1,5)	1 (1,4)	> 0.05
Localization (FIGOclassification)				> 0.05
Taype (3,5)	42 (65.6)	43 (65.2)	40 (66.7)	
Taype (4)	22 (34.4)	23 (34.8)	20 (33.3)	
CS indications				
Previous CS	15 (23.4)	13 (19.7)	20 (33.3)	> 0.05
Abnormal presentation	10 (15.6)	12 (18.2)	16 (26.7)	> 0.05
Fetal distress	5 (7.8)	8 (12.1)	10 (16.7)	> 0.05
Cephalo pelvic				> 0.05
Disproportion	10 (15.6)	11 (16.7)	9 (15)	< 0.001
Social factors	24 (37.5)	22 (33.3)	5 (8.3)	

The intraoperative and postoperative outcomes for the Study Group and control group are summarized in Table 2. Operation time, postoperative exhaust time, pre- and post-operative haemoglobin drop, intraoperative blood loss were all more than those of the control group in the study group (68.65 ± 11.87 vs 56.17 ± 9.18 min, 21.04 ± 4.98 vs 17.03 ± 1.3 h, 1.27 ± 0.59 vs 1.09 ± 0.43 g/dl, 613 ± 221 vs 532 ± 156 ml, *P* < 0.001, *P* < 0.001, *P* = 0.025, *P* = 0.011). There were no significant differences in the incidence of postpartum haemorrhage, transfusion, uterine artery ligation, intrauterine adhesion, 5 min Apgar score and asphyxia between the two groups (*P* > 0.05).

**Table 2:** Intraoperative and postoperative outcomes for caesarean myomectomy and Simple caesarean

	Study group (n = 130)	control group (n = 60)	*P*
Operation time (min)	68.65 ± 11.87	56.17 ± 9.18	< 0.001
Postoperative exhaust time (h)	21.04 ± 4.98	17.03 ± 1.3	< 0.001
Blood loss (ml)	613 ± 221	532 ± 156	0.011
Δ Hemoglobin level (g/dl)	1.27 ± 0.59	1.09 ± 0.43	0.025
Postoperative fever	8 (6.2)	3 (5)	> 0.05
Postpartum haemorrhage	12 (9.2)	2 (3.3)	> 0.05
Transfusion	8 (6.2)	1 (1.7)	> 0.05
Uterine artery ligation	6 (4.6%)	1 (1.7)	> 0.05
Intrauterine adhesion	0 (0)	0 (0)	> 0.05
Duration of lochia (d)	35.37 ± 2.7	36.1 ± 3.11	> 0.05
5-min Apgar score	9.96 ± 0.19	9.97 ± 0.18	> 0.05
Asphyxia	0 (0)	0 (0)	> 0.05

The intraoperative and postoperative outcomes in the different types of fibroids are summarized in Tables 3 and 4. For type III and V fibroids, the time of myoma removal, postoperative exhaust and pre- and post-operative haemoglobin drop and intraoperative blood loss in study group A were less than those in study group B (18.02 ± 3.89 vs 20.19 ± 5.32 min, 18.83 ± 2.57 vs 23.93 ± 6.84 h, 600 ± 194 vs 730 ± 277 ml, 1.20 ± 0.57 vs 1.59 ± 0.70 g/dl, *P* = 0.036, *P* < 0.001, *P* = 0.014, *P* = 0.008) (Table 3); For type IV uterine fibroids, only postoperative exhaust time was less in Study Group A than in Study Group B (19.27 ± 2.2 vs 21.35 ± 3.23 h, *P* = 0.016). However, there were no significant differences in intraoperative blood loss and the time of myoma removal (Table 4).

**Table 3:** Intraoperative and postoperative outcomes for type III and V fibroids

	Time of myoma resection (min)	Blood loss (ml)	Δ Hemoglobin level (g/dl)	Postoperative exhaust time (h)
Study group A (n = 42)	18.02 ± 3.89	600 ± 194	1.20 ± 0.57	18.83 ± 2.57
Study group B (n = 43)	20.19 ± 5.32	730 ± 277	1.59 ± 0.70	23.93 ± 6.84
*P*	0.036	0.014	0.008	< 0.001

**Table 4:** Intraoperative and postoperative outcomes for type IV fibroids

	Time of myoma resection (min)	Blood loss (ml)	Δ Hemoglobin level (g/dl)	Postoperative exhaust time (h)
Study group A (n = 22)	17.95 ± 2.38	520 ± 73	1 ± 0.2	19.27 ± 2.2
Study group B (n = 23)	18.13 ± 2.83	508 ± 131	1.03 ± 0.33	21.35 ± 3.23
*P*	> 0.05	> 0.05	> 0.05	0.016

Differences in the time of myomectomy according to the sequence of uterine incision suturing and myomectomy are presented in Table 5. The time of myoma removed was less in study group B1 than in study group B2 (18.24 ± 4.53 vs 20.7 ± 4.59 min, *P* = 0.033). However, there were no significant differences in intraoperative blood loss and the pre- and post-operative haemoglobin drop.

**Table 5:** Differences in intraoperative outcomes according to the sequence of uterine incision suturing and myomectomy

	Time of myoma resection (min)	Blood loss (ml)	Δ Hemoglobin level (g/dl)
Study group B1 (n = 33)	18.24 ± 4.53	634 ± 263	1.36 ± 0.76
Study group B2 (n = 33)	20.7 ± 4.59	671 ± 256	1.42 ± 0.65
*P*	0.033	> 0.05	> 0.05

## Discussion

Myomectomy during cesarean section has its unique advantages. During pregnancy, the myometrium becomes more elastic, the psuedocapsule of the myoma increases, the boundary between the myoma and the surrounding tissue is clearer, and the myoma is easier to peel [8, 13]. Along with postpartum uterine involution, myoma incision shrinks and myofibrils contract to compress blood vessels to accelerate hemostasis [8, 13, 14]. Myomectomy during cesarean section causes less damage to the myometrium and better protects fertility [15, 16]. A recent Mate analysis by Huang et al. [17], concluded that the removal of intramural myoma or multiple myomas greater than 7 cm in diameter during cesarean section increases intraoperative bleeding and prolongs the operation time, However, as long as appropriate hemostatic measures are adopted and implemented by experienced obstetricians and gynecologists, it is safe and feasible regardless of the size and location of the myoma. In this study, the operation time, intraoperative blood loss and postoperative exhaust time were significantly greater in the study group than in the control group, but there were no significant differences in the postpartum bleeding rate, blood transfusion rate and uterine artery ligation rate between the two groups, and there were no pronounced differences in the 5-min Apgar score and asphyxia rate of newborn. And it was further confirmed that large intramural myoma resection performed at the same time as cesarean section was safe and did not cause adverse effects on maternal and infant health.

The key to the safe removal of uterine myoma during cesarean section is to reduce intraoperative bleeding, which can be achieved by localizing the blood supply to the myoma or by blocking the uterine blood supply. For example, U-shaped suture [18]; intrathecal myomectomy proposed by Tinelli [19]; resection of myoma after blocking the blood supply to the base of the myoma by purse-string suture proposed by Lee et al. [14]; and ligation of the ascending branch of uterine artery combined with local injection of vasopressin into myoma [20] have achieved good surgical results. However, some of these methods are surgically skillful and some are only applicable to smaller interstitial myomas. The endometrial pathway was used to resect type III and type V uterine fibroids, and the results showed that the time of myomectomy, intraoperative bleeding, pre- and postoperative hemoglobin difference, and postoperative anal venting time was significantly less than that of serosa. Hatirnaz et al. [21] also reported similar research results, confirming that EM can effectively shorten the duration of myomectomy and reduce intraoperative bleeding. However, in type IV uterine myoma, close to the serous layer of the uterus, EM may damage more myometrium, and the time of myoma resection and intraoperative bleeding is not significantly reduced, but the postoperative exhaust time is significantly shorter. The ascending branch of the uterine artery branches out many arcuate arteries that penetrate the serosa and myometrium of the uterus, and send radial arterial branches into the endometrium, which in turn branch out the basilar and spiral arteries of the endometrium [2, 22]. Therefore, SM may damage the larger branch blood vessels of uterine artery and affect the blood perfusion of uterine tissue around the myoma, which may easily lead to difficult hemostasis or even refractory bleeding, and is also unfavorable to uterine involution during puerperium. In contrast, EM may only damage the spiral artery and contract rapidly as the uterus recovers, greatly reducing the risk of refractory postpartum hemorrhage. With the enlargement of the uterus during pregnancy, myoma indicates an increase in the area of the serosa. SM requires a large surgical incision, but EM results in a smaller surgical incision and further reduction of the incision as the uterus recovers. SM reduces the incision of the uterine serosa, decreases the incidence of postoperative pelvic adhesion and intestinal adhesion, and facilitates to the protection of fertility function [23, 24]. Does EM avoid adhesion between the serosa and surrounding organs, causing adhesion of the uterine cavity? Şafak Hatırnaz et al. [24] that intrauterine ultrasonography performed at 6 weeks postpartum in 118 patients who underwent the EM procedure did not reveal intrauterine adhesions, probably because the endometrial layer peeled off during the puerperium to rejuvenate avoiding the formation of intrauterine adhesions. In our study, no intrauterine adhesions were found in 64 patients with EM, and no intrauterine adhesions were found by B-mode ultrasonography at 42 days after delivery.

EM is indicated for myoma close to the uterine incision, and if the myoma is close to the bottom of the uterus, the difficulty of this procedure will be greatly increased. In this study, myomas near the bottom of the uterus were resected via SM. The traditional surgical procedure is to suture the uterine incision first, and then treat the uterine myoma. This is because suturing the uterine incision first to restore the integrity of the uterus reduces bleeding from the incision and promotes uterine contraction to reduce intraoperative bleeding, but problems such as penetration of the uterine cavity and difficulty in hemostasis occur when dealing with deep, large interstitial myomas. Under the premise that the tourniquet temporarily blocks the blood supply of the uterus, this study first resects the uterine myoma, and then sutures the uterine incision. It is the result of many years of practice and exploration by our team, and there is no literature report in China at present. The concept is that removing the uterine myoma through the uterine serous layer before closing the uterine incision can push the leiomyoma from the endometrial layer to the serous layer, make the uterine myoma pseudocapsule close to the serous layer, and reduce damage to the uterine myoma during myoma resection. Myomectomy guided by the uterine cavity avoids penetration of the uterine cavity, and prevents the occurrence of dead space, with more convenient and rapid resection of uterine myoma. In this study, this was also confirmed by the significantly shorter resection time of B1 myoma in the study group. Because the uterine blood supply is temporarily blocked with a tourniquet before myomectomy, to reduce bleeding from the uterine incision and the placenta dissection surface during myomectomy. And this was confirmed by the lack of significant differences in intraoperative bleeding and the difference in hemoglobin before and after surgery in study groups B1 and B2 in this study.

## Conclusions

To sum up, resection of interstitial myomas greater than 8 cm in diameter during hysterectomy is safe and feasible, and EM has the advantage of shortening the operative time and reducing intraoperative bleeding. The resection of uterine myoma through serosa, with the removal of the myoma first and then the suturing of the uterine incision, can shorten the time of myomectomy and is worth promoting. As a retrospective analysis, although the preoperative data have been controlled consistently in all groups of patients, it is still inevitable that some of the biases cannot be eliminated. The long-term recovery of hysteromyoma resection during cesarean section and the obstetric outcome of re-pregnancy need to be further studied.

## Data Availability

The research data used to support the fndings of this study were supplied by Ms. Shi under license and so cannot be made freely available. Requests for access to these data should be made to Ms. Shi.

## Abbreviations

EM: Endometrial myomectomy
SM: Serosal myomectomy
CS: Cesarean section
